# A Magnetostrictive Composite-Fiber Bragg Grating Sensor

**DOI:** 10.3390/s100908119

**Published:** 2010-08-30

**Authors:** Sully M. M. Quintero, Arthur M. B. Braga, Hans I. Weber, Antonio C. Bruno, Jefferson F. D. F. Araújo

**Affiliations:** 1 Department of Mechanical Engineering, Pontifical Catholic University of Rio de Janeiro, Rua Marquês de São Vicente 225, Gávea 22453-900, Rio de Janeiro, RJ, Brazil; E-Mails: sully@aluno.puc-rio.br (S.M.M.Q.); hans@puc-rio.br (H.I.W.); 2 Department of Physics, Pontifical Catholic University of Rio de Janeiro, Rua Marquês de São Vicente 225, Gávea 22453-900, Rio de Janeiro, RJ, Brazil; E-Mails: acbruno@puc-rio.br (A.C.O.B.); jferraz@fis.puc-rio.br (J.F.D.F.A.)

**Keywords:** magnetic field sensor, fiber Bragg grating, magnetostrictive composite

## Abstract

This paper presents a light and compact optical fiber Bragg Grating sensor for DC and AC magnetic field measurements. The fiber is coated by a thick layer of a magnetostrictive composite consisting of particles of Terfenol-D dispersed in a polymeric matrix. Among the different compositions for the coating that were tested, the best magnetostrictive response was obtained using an epoxy resin as binder and a 30% volume fraction of Terfenol-D particles with sizes ranging from 212 to 300 μm. The effect of a compressive preload in the sensor was also investigated. The achieved resolution was 0.4 mT without a preload or 0.3 mT with a compressive pre-stress of 8.6 MPa. The sensor was tested at magnetic fields of up to 750 mT under static conditions. Dynamic measurements were conducted with a magnetic unbalanced four-pole rotor.

## 1. Introduction

There are several instances where the measurement of magnetic fields is of interest for applications in machine diagnostics. In a hydroelectric generator, for example, monitoring magnetic fields is a requirement for performance prediction. A perfectly balanced rotating machine should have poles with identical magnetic fields. Hence, detecting the reduction in magnetic flux density caused by interturn short-circuits in a pole may allow identification of a malfunction [1]. Other cases where the capability of measuring magnetic fields is important to assure the proper operation of the machine are those where unbalanced forces are introduced in a rotating generator. Unbalancing may be caused by a defective bearing, manufacturing defects, or an unbalanced mass, all of them sources of non uniform magnetic field distribution in the air gap [2], which is the space between rotor and stator. Off-center or out-of-round conditions reduce operating efficiency and, in more severe cases, may lead to damage caused by magnetically induced heating or a rotor-to-stator rub. Therefore, monitoring the magnetic field in generators may be a valuable tool in order to provide early warnings of faults and prevent unscheduled maintenance stops or shutdowns.

In the environment that generators in hydroelectric plants operate, where high magnetic fields are present and limited space for installation prevent the use of most conventional sensors, Fiber Bragg gratings (FBG) offer a number of advantages over other magnetic field or electric current sensing technologies. Due to their immunity to electromagnetic interference, small size, corrosion resistance, and capability to perform remote, direct and absolute measurements, they are ideally suited to such severe operating conditions. There are also considerable benefits in exploring the large multiplexing capability of FBG-based sensors in these applications. The use of a large number of sensors along a single fiber link reduces installation costs with cabling and improves system reliability by allowing simultaneous monitoring of different operating parameters. Having that for motivation, the present paper introduces a compact FBG-based sensor that can be employed to measure DC and AC magnetic fields. The FBG is covered with a thick coating layer of a magnetostrictive composite that deforms due to changes in its magnetization state. Strains in the coating are transferred to the FBG. Through a calibration equation, their measurement may then be correlated to the acting magnetic field. The proposed device can also function as a displacement sensor, indicating the position of a magnet attached to an object moving with respect to the FBG coated with the magnetostrictive composite.

Magnetostrictive composites made of Terfenol-D particles embedded in a resin matrix have attracted considerable attention due to the improvements they offer over the bulk material performance. They exhibit higher electric resistivity, extended frequency response, superior tensile strength, better durability, and greater flexibility in shape and form than the monolithic Terfenol-D. Magnetostrictive composites first came into play in the early 1990’s, when Sandlund *et al*. proposed a method of combining Terfenol-D particles with a non-metallic binder to form a composite [3]. Duenas and Carman [4] later demonstrated that Terfenol-D/epoxy composites can exhibit magnetostrictive strain responses comparable to that of the monolithic. Even though it has been previously demonstrated that bonding FBGs to monolithic Terfenol-D results in efficient sensors for different applications [5–10], there are very few published reports on the combined use of magnetostrictive composites and FBGs for sensing applications. In Reference [11], Liu *et al*. proposed a magnetic field sensor that employs a FBG bonded, not embedded, to a piece of Terfenol-D/epoxy composite. Their results have demonstrated the potential of using magnetostrictive composites in conjunction with FBGs in sensing magnetic fields. However, one may expect that a FBG bonded to the outer surface of a Terfenol-D/epoxy composite bar will be less protected from the surroundings, and prone to degradation, than one that has been embedded, particularly in the harsh environments often associated with industrial applications.

This article is divided in two parts. First, the focus is on the characterization of the magnetostrictive composite. It is shown that a composite with 30% volume fraction of Terfenol-D particles exhibits excellent magnetostrictive properties, even without compressive preloads or any specific particle orientation. Then, attention is turned to the proposed sensor, which is light and compact, having a diameter of 1.5 mm and length of 7 mm. Its static response was evaluated by subjecting the prototype to magnetic fields up to 750 mT. Dynamic tests were carried out in a laboratory rotor that presented a magnetic unbalance of approximately 7% in one of its four poles. Results were compared with a Hall Effect sensor showing excellent agreement.

## 2. Characterization of the Magnetostrictive Composite

Two different magnetostrictive coating layers for the fiber sensor were evaluated, both using Terfenol-D particles but different binders. In the first group, the matrix was a cycloaliphatic epoxy resin (AeroMarine 300/21) with Shore-D hardness ranging from 80 to 85. For the second, the binder was a polyurethane resin (AeroMarine Casting Resin), with Shore-D hardness of 75. Both materials are supplied by AeroMarine Products. With irregular shape and characteristic size ranging from 50 to 300 μm, the magnetostrictive particles of Terfenol-D, provided by Etrema Products, were separated into three classes according to Table 1.

In order to produce the test specimens for magnetic characterization of the composite, particles and resin were mixed and poured into cylindrical molds with diameter and height of 3 mm. Subsequently, the molds were repeatedly degassed to remove unwanted trapped air. The cast specimens were allowed to cure at room temperature for 48 hours before being removed from the mold. Four groups of specimens, each with three samples, were prepared according to Table 2.

**Figure 1.**
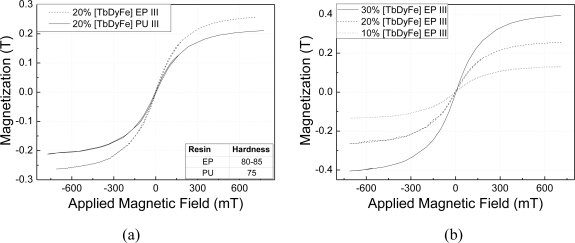
Magnetization *vs*. applied magnetic field for: **(a)** different binding resins (comparison between samples in Groups A and B); and **(b)** different volume fractions of Terfenol-D (samples in Group C).

The relationship between the magnetization and applied magnetic field for particle sizes larger than 200 μm (Class III) and volume fraction of 20%, is shown in Figure 1(a). Measurements clearly indicate that the magnetization of the composite prepared with the epoxy resin (EP) is 22% larger than that of those prepared with the polyurethane resin (PU). This result provides evidence of the effect of binder hardness on the magnetization response of the composite. Figure 1(b) compares magnetization curves of composites in Group C, with particulate of the same size (Class III) and volume fractions of 10, 20, and 30%, the latter presenting stronger response than the other two.

**Figure 3.**
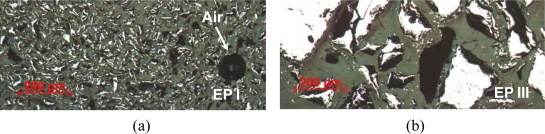
Cross section micrographs of specimens in Group D with particle sizes: **(a)** smaller than 50 μm (Class I); **(b)** larger than 200 μm (Class III).

Analyzing the microscope images in Figure 3, one observes that the Terfenol-D particles were fully bonded to the matrix but some trapped air was also found in the samples. The dark regions correspond to the binding matrix and the lighter ones to Terfenol-D particles.

## 3. Fiber Optic Magnetostrictive Sensor

**Figure 4.**
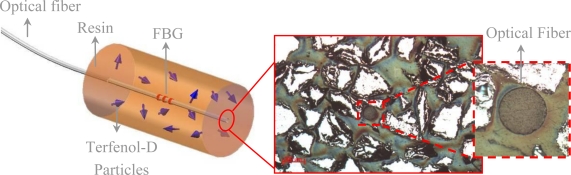
Schematic configuration of the magnetostrictive composite FBG sensor; the cross section micrograph in the inset shows Terfenol-D particles and the optical fiber.

The sensor prototype developed in this work is schematically depicted in Figure 4. It was constructed by covering the FBG inscribed in the optical fiber with a thick magnetostrictive coating. The chosen composite used a binding matrix of epoxy resin (EP), volume fraction of 30%, and Terfenol-D particles with sizes larger than 200 μm (Class III). Figure 4 also presents a cross section micrograph of one of the sensors, showing the optical fiber and the random distribution of Terfenol-D particles in the coating layer. The tested prototypes were cylindrical in shape, with a length of 7 mm and diameters ranging from 1.5 to 5.0 mm.

The sensor is based on Fiber Bragg Gratings (FBG), which consists of a periodic modulation in the core refractive index of a single-mode optical fiber along a small length of fiber, approximately 1 mm in this case. The FBG operates as a highly selective wavelength filter, which reflects a narrow band of light around its Bragg wavelength. The Bragg wavelength is related to the effective refractive index of the fiber core,*n*_eff_, and to the spatial periodicity in the index modulation, Λ [12]:

(1) $$\lambda_{B}=2n_{\text{eff}}\Lambda$$

The sensor employs a magnetostrictive coating as an actuator that, in response to changes in magnetic fields, strains the optical fiber containing one or more FBGs. The basic principle of measuring strains with FBG lies in monitoring wavelength shifts of the reflected Bragg-signal as a function of changes in the effective refractive index or spatial periodicity of the grating. The Bragg wavelength shift due to strain and temperature can be expressed as:

(2) $$\Delta \lambda_{B}=\lambda_{B}[(\alpha_{f}+\zeta_{f})\Delta T+(1-p_{e})\Delta \varepsilon ]$$

where *α_f_* is the thermal expansion coefficient of the fiber, *ζ_f_* its thermo-optical coefficient, and *p_e_* the optical fiber strain-optical coefficient, which is 0.22 for a germanosilicate glass. In the absence of temperature changes, it is possible to measure strain from the wavelength shift as [12]:

(3) $$ɛ=1.28\frac{\Delta \lambda_{B}}{\lambda_{B}}$$

### 3.1. Static Test Results

A small electromagnet was used to apply a uniform magnetic field over the sensor whose axis was aligned to the axis of the electromagnetic poles. The peak wavelength shift due to the applied magnetic field, which is related to the strain response of the sensor, was observed using a commercial optical spectrum analyzer (Micron Optics sm125). No spectral distortion of the reflected wavelength spectrum, which might otherwise be associated with birefringence induced by transverse strains in the embedded FBG sensor, was observed.

**Figure 5.**
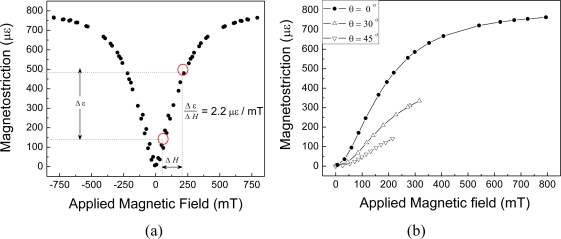
**(a)** Sensor response without a preload; **(b)** Sensor sensibility changes with the angle between sensor axis and the applied magnetic field. Results are for sensor with length of 7 mm and 1.5 mm diameter.

Figure 5(a) shows the magnetostrictive sensor response without an applied compressive preload. Measurements were performed under controlled temperature conditions, at 23 ± 0.5 °C. No warm up of the sensors were observed during calibrations. The particles in the coating layer were randomly oriented. Response is approximately linear up to 250 mT. The sensitivity may be expressed as the ratio Δ*ε* / Δ*H*, where Δ*ε* is the strain induced in the FBG, related to the Bragg wavelength shift through Equation (3), and Δ*H* is he applied magnetic field. For this sensor configuration, the estimated sensitivity was of 2.2 × 10^−6^ mT^−1^. Employing a FBG interrogation system with strain resolution of 0.8 × 10^−6^, which may be achieved by many of the available commercial interrogators, leads to a minimum detectable change in the electromagnetic field of 0.4 mT. Angle dependency to the magnetic field was also measured and results, shown in Figure 5(b), indicate that sensibility decreases 1.3% when sensor is rotated 1° with respect to the orientation of the magnetic field. Temperature cross-sensitivity was also evaluated. Measurements revealed a temperature sensitivity of 44 pm/°C, which, in terms of apparent strain, corresponds to 36 × 10^−6^ C^−1^. This high cross-sensitivity may be compensated by employing a second FBG, not coated by the magnetostrictive composite, and detecting only temperature variations.

**Figure 6.**
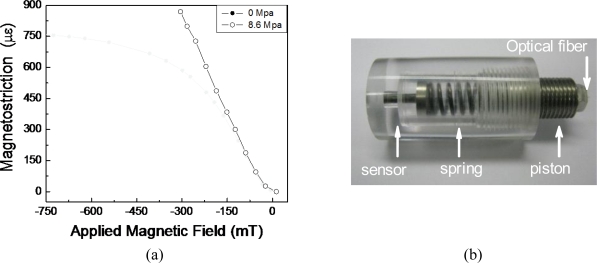
**(a)** Comparison of sensor response with and without a compressive preload. In both cases, results are for sensor with length of 7 mm and 1.5 mm diameter. **(b)** Load cell developed to apply the preload.

It is well known that compressive mechanical preloads modify the strain response of magnetostrictive materials [13,14]. They do so by changing the slope and maximum strain in the characteristic magnetostrictive curve (strain *vs*. applied field). In order to evaluate the effect of compressive preloads on the sensor response, a load cell was employed to produce a compressive pre-stress of 8.6 MPa along the axial direction of one of the prototypes (Figure 6). When the compressive pre-stress of 8.6 MPa was applied, as shown in Figure 6(a), sensibility increased to 36 × 10^−6^ mT^−1^, a value 40% higher than that without the compressive pre-stress. Further, with the preload, sensor response is approximately linear up to the highest tested value of the magnetic field (300 mT). Higher values were not tested due to experimental setup limitations.

To optimize the sensor design and better understand how size affects its performance, prototypes with 5.0, 3.0, and 1.5 mm diameters were tested, all 7.0 mm long and with equal volume fraction (30%) of Terfenol-D particles. In every case, the magnetostriction outputs for all prototypes were indistinguishable, implying a non-dependence on the geometry and also indicating homogeneous strain distribution within the magnetostrictive coating layer. These results suggest that sensitivity does not depend on the sensor diameter.

### 3.2. Dynamic Test Results

**Figure 7.**
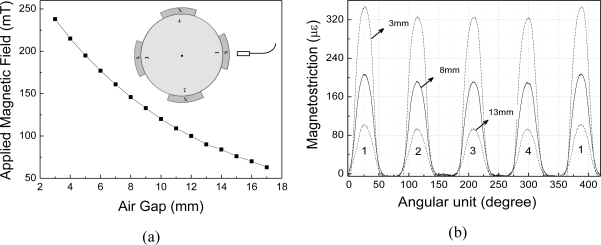
**(a)** Dependence of the radial component of the magnetic field on the distance from the pole surface number 1 (inset depicts the setup); **(b)** angular magnetic field distribution for different air gaps.

The sensor dynamic response was evaluated by applying an AC magnetic field produced by four permanent magnets attached to a rotor spinning at 504 rpm. A schematic representation of the rotor is presented in the inset of Figure 7(a). The adhesive layer employed to fix pole number 1 was slightly thicker than the other three, resulting in a magnetic unbalance of approximately 7% between this pole and the others.

The test involved measuring the magnetic flux in the air gap to detect and measure the magnetic unbalance in the poles. Each rotor pole induced a strain that was proportional to the change in flux as the pole swept through the sensor axis. The peak wavelength shift of the sensor due to induced magnetic field has been measured using a commercial, dynamical, optical spectrum analyzer (Micron Optics sm130) acquiring data at 1.0 kHz. The sensor has presented a stable and reproducible response through several cycles of the rotor.

The intensity of the magnetic field in the radial direction for pole 1 at different air gaps is shown in Figure 7(a). Figure 7(b) shows the magnetic field distribution while the rotor rotates. As each pole passes, there is a peak in the induced signal. Each peak of the waveform represents the peak flux across one rotor pole. The difference between the maximum of peak 1 and the peaks 2, 3 and 4, for the three air gaps analyzed (3 mm, 8 mm and 13 mm), correspond to the induced magnetic unbalance.

The asymmetry of the magnetic field may be noticed for the three air gaps analyzed. In the case of the 13 mm air gap, the difference between the two maxima is 7.8 × 10^−6^, and it is still possible to measure the magnetic unbalance in spite of the proximity to the resolution limit of the dynamic FBG interrogator, which was of approximately 5 × 10^−6^.

**Figure 8.**
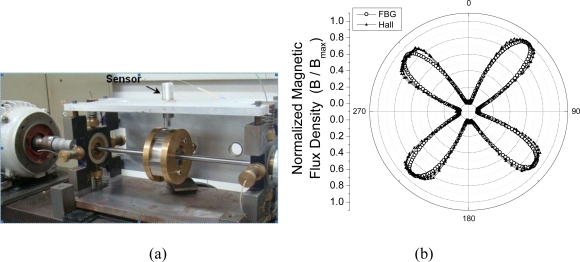
**(a)** Experimental setup; **(b)** Synchronous electrical and optical measurement of magnetic field from each pole swept passing the sensors.

Finally, the response of the optical sensor based on FBG was compared with a conventional electric magnetic sensor based on Hall Effect. Figure 8(b) shows the normalized signal measured by both sensors. There was an intrinsic difference of approximately 7% between the four magnetic poles being monitored, with the FBG magnetic sensor being comparable to the electric sensor in its ability to detect a change in the magnetic field.

## 4. Conclusions

In the presented work, magnetic properties of eight different magnetostrictive composites were investigated. The influence of resin hardness, volume fraction, and particle size of Terfenol-D on the composites’ magnetostrictive response was evaluated. Among the tested combinations, the optimal composition was obtained by using the harder epoxy resin (80-85 Shore-D), 30% volume fraction, and larger Terfenol-D particle sizes (200–300 μm).

A compact magnetostrictive composite fiber Bragg grating sensor, cylindrical in shape with 1.5 mm diameter and 7 mm length has been demonstrated and tested in both static and dynamic conditions. Sensitivity of 2.2 × 10^−6^mT^−1^ was obtained using a FBG interrogation system with strain resolution of 0.8 × 10^−6^. In this case, the lower detectable change in magnetic field is of 0.4 mT.

The sensitivity of the present sensor is approximately 50% lower than that reported in [6] by Mora *et al*. using monolithic Terfenol-D. Although presenting a lower sensibility, the sensor demonstrated here is lighter and smaller than the one in [6]. Furthermore, it has been shown that by applying a compressive preload and generating a pre-stress of 8.6 MPa, sensibility and linearity range of the present sensor increases by 40% and 20% respectively. With this preload, the sensor resolution is improved to 0.3 mT.

Although it was not shown here, the sensor can be multiplexed and temperature compensated. Indeed, only the small length of the fiber where the FBG has been inscribed needs to be coated with the magnetostrictive composite. Thus, several FBGs, individually coated, may share the same optical fiber with temperature sensors or other of FBG-based sensors.

Among other applications, the sensor has been designed to be employed in monitoring the air gap in electric generators. Comparison of dynamic measurements in a small, magnetically unbalanced rotor, performed with both the proposed sensor and a conventional Hall Effect sensor were consistent, exhibiting very good agreement.

## Figures and Tables

**Figure 2.**
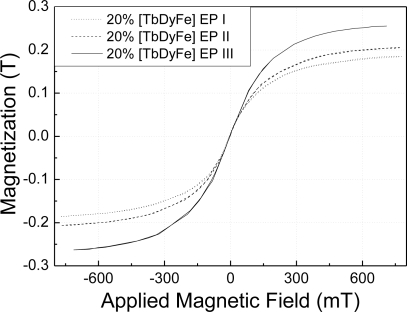
Magnetization of magnetostrictive composites with different particle sizes.

**Table 1.** Particle size distribution.

Class	Particle Size (μm)
I	<50
II	74–150
III	>200

**Table 2.** Specimens for magnetic characterization.

**Group**	**Particle Size^(a)^**	**Resin^(b)^**	**Volume Fraction**
A	Class III	EP	20%
B	Class III	PU	20%
C	Class III	EP	10%, 20% and 30%
D	Class I, II and III	EP	20%

Notes:

(a) According to Table 1;

(b) EP: Epoxy Resin, PU: Polyurethane Resin

## References

[1] Lalonde F (1992). Magnetic Field Measurement, Hydropower’92.

[2] Talas P, Toom P (1983). Dynamic measurement and analysis of air-gap variations in large hydroelectric generators. IEEE Trans. Power App. Syst.

[3] Sandlund L, Fahlander M, Cedell T, Clark AE, Restorff JB (1994). Magnetostriction, elastic moduli, and coupling factors of composite Terfenol-D. J. Appl. Phys.

[4] Duenas TA, Carman GP (2001). Particle distribution study for low-volume fraction magnetostrictive composites. J. App. Phys.

[5] Chiang KS, Kancheti R, Rastogi V (2003). Temperature-compensated fiber-Bragg-grating-based magnetostrictive sensor for dc and ac currents. Opt Eng.

[6] Mora J, Diez A, Cruz JL, Andrés MV (2000). A magnetostrictive sensor interrogated by fiber gratings for DC-current and temperature discrimination. IEEE Photon. Tech. Lett.

[7] Satpathi D, Moore JA, Ennis MG (2005). Design of a Terfenol-D based fiber optic current transducer. Sensor. J. IEEE.

[8] Carvalho HR, Bruno AC, Brag AM, Valente LCG, Triques ALC, Caspary MC (2007). Remote magnetostrictive position sensors interrogated by fiber Bragg gratings. Sens. Actuat. A.

[9] Ambrosino C, Campopiano S, Cutolo A, Cusano A (2008). Sensitivity tuning in Terfenol-D based fiber bragg grating magnetic sensors. Sensor. J. IEEE.

[10] Ambrosino C, Capoluongo P, Campopiano S, Cutolo A, Giordano M, Davino D, Visone C, Cusano A (2007). Fiber bragg grating and magnetic shape memory alloy: Novel high-sensitivity magnetic Sensor. Sensor. J. IEEE.

[11] Liu HL, Tam HY, Lo CY, Or SW (2007). A Novel High Frequency Magnetostrictive Composite-Fiber Bragg Grating Sensor.

[12] Othonos A, Kalli K (1999). Fiber Bragg Gratings: Fundamentals and Applications in Telecommunications and Sensing.

[13] Armstrong W (2002). A general magneto-elastic model of Terfenol-D particle actuated composite materials. J. Intell. Mater. Syst. Struct.

[14] Goran E (2000). Handbook of Giant Magnetostrictive Materials.

